# Aflatoxin B1: from metabolism to multi-organ carcinogenesis

**DOI:** 10.17179/excli2026-9793

**Published:** 2026-10-05

**Authors:** Pan Yu, Jiacheng Li, Weiya Cao, Yongchang Cao, Wenhui Lv, Hanxu Wang, Jiaxiang Zhang, Xiaoyu Bai, Shuhui Feng

**Affiliations:** 1 The First Affiliated Hospital of Anhui University of Science and Technology (Huainan First People's Hospital), Huainan 232001, China; 2 College of Public Health, Anhui University of Science and Technology, Hefei 230000, China; 3 Joint Research Center of Occupational Medicine and Health, Institute of Grand Health, Hefei Comprehensive National Science Center, Hefei 230000, China; 4 College of Life and Health, West Anhui University, Luan, Anhui, China

**Keywords:** Aflatoxin B1, hepatocellular carcinoma, cytochrome P450, DNA adducts, TP53, aryl hydrocarbon receptor, oxidative stress, epigenetics

## Abstract

Aflatoxin B1 (AFB1), a Group 1 carcinogen classified by the International Agency for Research on Cancer, chronically exposes a substantial proportion of the global population through dietary intake, primarily from fungal contamination of staple crops such as maize and peanuts. Climate change is progressively expanding this problem beyond its traditional tropical and subtropical boundaries into temperate regions of Europe and Asia. The carcinogenicity of AFB1 is initiated by its metabolic activation via hepatic cytochrome P450 enzymes, yielding AFBO, which forms DNA adducts and subsequently induces the signature G-to-T transversion mutation at codon 249 of TP53. However, recent evidence indicates that this canonical pathway represents only part of a far more complex picture. This review advances three interconnected themes. First, the aryl hydrocarbon receptor (AHR) has been established as an upstream regulatory hub of AFB1 toxicity, both driving CYP-mediated metabolic activation and mediating PD-L1 upregulation to promote immune evasion. Second, oxidative stress, epigenetic reprogramming, and autophagic dysfunction operate as accelerators that drive malignant transformation. Third, the multi-organ toxicity of AFB1, encompassing intestinal barrier disruption, systemic immunosuppression, and renal and reproductive toxicity, together with its co-exposure with hepatitis B virus and other mycotoxins, is expanding our traditional understanding of synergistic carcinogenesis. On the translational front, this review integrates the biomarker continuum from exposure (AFB1-albumin adducts) through effect (TP53 mutation, epigenetic marks) to susceptibility (GST/CYP genotypes, GNMT, CDKN2A), and synthesizes a multi-tiered intervention framework spanning source control, chemoprevention (AHR inhibitors, antioxidants), microbial/enzymatic detoxification, and precision immunotherapy (Figure 1(Fig. 1)).

## 1. Introduction

### 1.1 AFB1 contamination, exposure, and disease burden

Aflatoxins are secondary metabolites produced by Aspergillus flavus and Aspergillus parasiticus (Cao et al., 2022[5]; Rushing and Selim, 2019[50]). Over 18 congeners have been identified, among which AFB1 exhibits the most potent toxicity and carcinogenicity (Li et al., 2022[26]; Wang et al., 2025[68]). The molecule possesses a difuranocoumarin skeleton that confers exceptional thermal stability, rendering conventional food processing inadequate for its complete elimination and thereby permitting its persistence throughout the entire food supply chain (Benkerroum, 2020[2]).

Global surveillance data consistently demonstrate elevated detection rates and concentrations of AFB1 in maize, peanuts, tree nuts, cottonseed, and spices (Lemmetty et al., 2025[24]; Rushing and Selim, 2019[50]). Of particular concern is the shifting contamination landscape: climate change-driven alterations in temperature and humidity patterns are extending AFB1 contamination from traditional endemic zones into temperate regions of Europe and Asia (Lemmetty et al., 2025[24]), steadily expanding the population at risk.

Acute AFB1 poisoning manifests as hepatic necrosis, jaundice, and edema, and can be rapidly fatal (Benkerroum, 2020[2]). Yet of greater public health significance is chronic low-level exposure, which drives hepatocellular carcinoma (HCC) development, suppresses immune function, and impairs childhood growth and development (da Silva et al., 2021[10]; Rushing and Selim, 2019[50]). The International Agency for Research on Cancer has classified AFB1 as a Group 1 human carcinogen (Ostry et al., 2017[45]). The highest-risk regions are those where dietary AFB1 exposure overlaps with areas of high hepatitis B virus (HBV) endemicity, notably sub-Saharan Africa and Southeast Asia. Under this co-exposure scenario, HCC risk is elevated 30- to 60-fold relative to either exposure alone (Jin et al., 2023[20]; Xing et al., 2025[76]). Children are particularly vulnerable, as their developing immune and metabolic systems exhibit heightened sensitivity to AFB1 toxicity (da Silva et al., 2021[10]; Ghantous et al., 2021[15]).

### 1.2 The classical paradigm and its limitations

Decades of research have established a well-defined framework: hepatic cytochrome P450 1A2** (**CYP1A2) and hepatic cytochrome P450 3A4 (CYP3A4) oxidize AFB1 to the highly reactive electrophilic intermediate AFB1-8,9-exo-epoxide (AFBO) (Cao et al., 2022[5]; Gerdemann et al., 2023[14]). AFBO covalently binds to DNA guanine residues, generating AFB1-N⁷-guanine adducts, which either undergo spontaneous depurination or convert to more stable ring-opened formamidopyrimidine (Fapy) adducts (Fedeles and Essigmann, 2018[11]). When such lesions remain unrepaired or are misrepaired, G-to-T transversion mutations arise, with the hotspot mutation at tumor protein p53 (TP53) codon 249 (arginine to serine, R249S) serving as the molecular fingerprint of AFB1-associated HCC (Fedeles and Essigmann, 2018[11]; Link and Iwakuma, 2017[32]).

While this AFBO-DNA adduct-TP53 mutation axis possesses considerable explanatory power, it confronts three irreducible limitations. First, marked inter-individual variation in HCC susceptibility at equivalent exposure levels points to the profound involvement of host genetic, epigenetic, and environmental modifiers. Second, the protracted latency between exposure and HCC development implies that AFB1 must perturb broader cellular homeostatic mechanisms to accomplish malignant transformation. Third, the absence of TP53 codon 249 mutations in a subset of AFB1-associated HCCs indicates the existence of alternative oncogenic pathways.

Over the past decade, advances in high-throughput omics technologies and mechanistic investigations have progressively filled these gaps. AHR signaling, epigenetic reprogramming, oxidative stress signaling, autophagic dysregulation, and immunosuppressive microenvironment remodeling, a constellation of non-canonical pathways, have been successively identified. However, these discoveries remain scattered across subfields, lacking a unified framework for understanding their interrelationships.

### 1.3 Scope and positioning 

This review aims to integrate key literature from the past decade to construct a network model of AFB1 hepatocarcinogenesis that transcends the classical gene mutation paradigm. The central thesis is that AFB1 carcinogenicity results from the synergistic interplay between genotoxic injury (the seed) and microenvironmental remodeling (the soil). The aryl hydrocarbon receptor (AHR) is a ligand-activated transcription factor and master molecular regulatory node bridging xenobiotic metabolic responses and immune signalling, and it serves as the molecular switch connecting these two dimensions (genotoxic injury and microenvironmental remodeling).

Specifically, this review will: (1) update current understanding of AFB1 metabolic activation and detoxification, with emphasis on determinants of individual susceptibility; (2) construct a multidimensional carcinogenesis model encompassing genotoxicity, oxidative stress, epigenetics, autophagy, and immune evasion; (3) elucidate the molecular basis of AFB1-HBV synergy while incorporating multi-organ toxicity and combined toxicity into the framework; (4) critically evaluate the current state of biomarkers and risk stratification; and (5) map the translational roadmap from prevention to therapeutic intervention (Figure 2(Fig. 2)).

## 2. AFB1 Metabolic Activation and Detoxification

The toxic consequences of AFB1 depend on the net balance between phase I activation and phase II detoxification. This equilibrium, modulated by genetic polymorphisms, species differences, and environmental factors, fundamentally determines individual and species susceptibility.

### 2.1 Phase I metabolism: activation

AFB1 is intrinsically relatively inert and requires bioactivation to exert its carcinogenic effects. Hepatic cytochrome P450 enzymes catalyze epoxidation of the terminal furan ring to generate AFBO (Cao et al., 2022[5]; Gerdemann et al., 2023[14]).

In human liver, CYP1A2 and CYP3A4 constitute the principal mediators of AFB1 activation (Gerdemann et al., 2023[14]; Iori et al., 2024[19]). CYP3A4, the most abundant hepatic P450 isoform, simultaneously generates both AFBO and the hydroxylated metabolite aflatoxin Q1 (AFQ1). CYP1A2, though expressed at lower levels, exhibits higher catalytic efficiency at low substrate concentrations. Recent investigations have identified additional isoforms in extrahepatic tissues and animal models. CYP1A1 participates in intestinal AFB1 activation, potentially contributing to localized DNA damage and barrier dysfunction (Iori et al., 2024[19]). In cattle, CYP3A74 represents the major contributor to AFBO formation, with knockout experiments confirming its critical role in cytotoxicity (Iori et al., 2024[19]). Molecular docking and site-directed mutagenesis studies have further elucidated AFB1 and CYP1A2 interactions, demonstrating that Phe-125, Phe-226, and Phe-260 stabilize AFB1 binding through CH/π interactions (Wu et al., 2019[73]).

The stereochemical outcome of the epoxidation reaction carries decisive biological implications. AFB1 epoxidation generates both exo- and endo-isomers, yet only the exo-isomer possesses the configuration required for efficient covalent binding to DNA (Fedeles and Essigmann, 2018[11]). The endo-isomer undergoes rapid hydrolysis to the non-toxic AFB1-8,9-dihydrodiol.

AHR has been firmly established as the upstream regulatory hub of AFB1 metabolic activation. As a ligand-activated transcription factor, AHR, upon stimulation by AFB1 and its metabolites, transcriptionally upregulates CYP1A1 and CYP1A2 (Iori et al., 2024[19]; Mary et al., 2024[41]). This constitutes a feed-forward amplification loop, AFB1 activates AHR, which in turn enhances AFB1 activation. This AHR-CYP1A axis represents the first amplifier of AFB1 toxicity and a critical node for therapeutic intervention. Co-occurring mycotoxins, such as sterigmatocystin (VerA), can enhance AFB1 genotoxicity through CYP1A1 induction (Budin et al., 2021[3]). Given that AFB1 frequently co-occurs with its biosynthetic precursors in contaminated agricultural commodities, this synergistic enhancement warrants careful attention.

### 2.2 Phase II metabolism: detoxification

Elimination of AFB1 metabolites depends on phase II detoxifying enzymes. Glutathione S-transferases (GSTs), particularly GSTM1 and GSTT1, catalyze the conjugation of reduced glutathione with AFBO to generate water-soluble mercapturic acid derivatives for excretion (Gerdemann et al., 2023[14]; Thiendedsakul et al., 2022[60]). Genotypes lacking GSTM1 or GSTT1 are associated with elevated HCC risk in AFB1-exposed populations. UDP-glucuronosyltransferases (UGTs) enhance the water solubility of hydroxylated metabolites such as aflatoxin M1 (AFM1) and AFQ1 through glucuronidation (Manzini et al., 2017[39]). The relative contributions of these detoxification pathways vary considerably across species and among individuals.

The activation-detoxification balance provides a unifying framework for understanding AFB1 susceptibility (Cao et al., 2022[5]; Gerdemann et al., 2023[14]). Individuals exhibiting high CYP activity coupled with low GST capacity, whether due to genetic polymorphisms, environmental induction, or pathological states, face elevated risk of AFB1 toxicity and HCC. Conversely, the opposite configuration confers relative protection. The practical value of this framework lies in its capacity to identify high-risk individuals through functional phenotyping or genotyping of key metabolic enzymes, enabling targeted surveillance and prevention (Figure 3(Fig. 3)).

### 2.3 Species differences

Species differences in AFB1 metabolism profoundly influence animal model selection and interpretation of findings. Systematic comparisons of mouse, rat, and human primary hepatocytes have revealed fundamental disparities (Gerdemann et al., 2023[14]). Mouse hepatocytes exhibit the highest metabolic rate, predominantly generating AFP1 and its glucuronide conjugates, with high efficiency of glutathione conjugation, explaining the marked resistance of mice to AFB1 hepatocarcinogenesis. Human hepatocytes primarily produce AFM1, AFQ1, and aflatoxicol, with limited glutathione conjugation capacity, accounting for high human susceptibility. Rat hepatocytes display the highest levels of DNA adducts, with intermediate susceptibility. Notably, despite the mechanistic insights gained from rodent and tree-shrew experimental models, inter-species metabolic divergence imposes inherent limitations; key mechanistic observations from pre-clinical models require subsequent human-based biomarker, cohort or clinical validation before translational implementation. These differences must be carefully considered when extrapolating data from rodent models to humans (Figure 4(Fig. 4)).

### 2.4 Extrahepatic metabolism and the gut microbiota

While the liver serves as the primary site of AFB1 metabolism, significant metabolic activity also occurs in the intestine, kidney, and lung (Gerdemann et al., 2023[14]; Wang et al., 2025[68]). Intestinal CYP1A1-mediated AFB1 activation can inflict local DNA damage and barrier dysfunction. Low-level renal metabolism contributes to AFB1-associated nephrotoxicity.

The gut microbiota has emerged as one of the most active frontiers in recent years (Lemmetty et al., 2025[24]; Yuan et al., 2025[80]). The microbial community possesses two opposing capacities, degradative detoxification of AFB1 on the one hand, and reactivation of conjugated metabolites to genotoxic species on the other. Gut microbiota of black soldier fly larvae cooperate with host detoxifying enzyme systems to degrade AFB1 (Yuan et al., 2025[80]). Lactic acid bacteria (Lactobacillus, Bifidobacterium) remove AFB1 through binding mechanisms, with contributions ranging from 16 % to 71 %, substantially exceeding enzymatic degradation, which accounts for only 0-18 % (Lemmetty et al., 2025[24]).

Recent investigations have extended the scope of gut microbiome research from in vitro models to ruminant rumen ecosystems. Li and colleagues (Li et al., 2025[27]) used integrated metagenomic, metaviromic and metabolomic analyses to show that AFB1 remodels the microbiome-virome-metabolome axis in goat rumen in a dose-dependent manner at exposure doses of 50 and 500 µg/kg. This toxic effect is reflected by reduced ruminal pH together with declines in total volatile fatty acids, acetate and propionate. It also causes repression of the fiber-degrading genera Pelagibacter and Flavobacterium, alongside the expansion of Cryptobacteroides. Seven carbohydrate-active enzymes (GT92, GT20, CE7, GT32, GT35, GT57, GT50) were more abundant in controls, suggesting impaired fiber degradation capacity. Metabolomics identified 1,197 differentially abundant metabolites, including the teratogenic cytochalasin and cell death-inducing chrysophanol. Elevated viral load in the high-dose group suggested that AFB1 may activate prophages, exacerbating microecological dysbiosis.

From a broader perspective, Mafe and Büsselberg (2026[36]) provided a systematic review regarding the bidirectional crosstalk between the microbiome and mycotoxins. Microbial enzymes including de-epoxidases, lactonases and reductases are involved in mycotoxin biotransformation. Probiotics strengthen epithelial barrier integrity and restore microecological homeostasis. They also modulate immune responses via toxin sequestration, competitive exclusion and anti-inflammatory actions mediated by the NF-κB, STAT3 and IL-6 signaling pathways. However, the detoxification efficacy of probiotics is strain-specific, necessitating targeted screening.

The gut microbiome therefore constitutes not only a determinant of individual susceptibility to AFB1 exposure but also a promising intervention target. Strategies rooted in microbiome research span from probiotic screening to fecal microbiota transplantation. These approaches are advancing from in vitro experimental models toward practical field deployment within ruminant production systems (Figure 5(Fig. 5)).

## 3. Multidimensional Mechanisms of AFB1 Hepatocarcinogenesis

The carcinogenic actions of AFB1 extend far beyond genotoxicity. They constitute a complex, multi-layered network of cellular and microenvironmental perturbations. The following sections examine these mechanisms across three dimensions: the trigger (genotoxic injury), the accelerators (oxidative stress, epigenetics, autophagy), and the soil (immune microenvironment).

### 3.1 The trigger: genotoxic injury and genomic instability

#### 3.1.1 AFB1-DNA adducts

The initiating event in AFB1 hepatocarcinogenesis is the covalent binding of AFBO to DNA, predominantly at the N⁷ position of guanine (Fedeles and Essigmann, 2018[11]; Theumer et al., 2018[59]). The AFB1-N⁷-guanine adduct is chemically unstable and may either undergo spontaneous depurination, generating apurinic sites that, if misrepaired, are mutagenic, or convert to the more stable ring-opened Fapy adduct (Fedeles and Essigmann, 2018[11]). Both lesions, if not faithfully repaired, give rise to G-to-T transversion mutations (Hu et al., 2026[17]; Theumer et al., 2018[59]). Molecular dynamics simulations have elucidated the structural recognition features of AFB1-DNA adducts and the mechanisms by which these lesions impede DNA replication and repair (Hu et al., 2026[17]).

#### 3.1.2 TP53 codon 249 mutation

The G-to-T transversion at TP53 codon 249 (R249S) constitutes the molecular hallmark of AFB1-associated HCC (Fedeles and Essigmann, 2018[11], Link and Iwakuma, 2017[32]). This mutation occurs at remarkably high frequency in HCCs from high-exposure regions (Jin et al., 2023[20]). R249S ablates the DNA-binding capacity of p53, resulting in complete loss of cell cycle checkpoint, DNA repair, and apoptosis-inducing functions (Link and Iwakuma, 2017[32]). Nevertheless, the absence of this mutation in a subset of AFB1-associated HCCs indicates that alternative pathways can bypass this event to drive oncogenesis.

#### 3.1.3 Disruption of DNA repair

AFB1 not only creates DNA damage but also actively subverts the repair systems. Key proteins in the nucleotide excision repair (NER), base excision repair (BER), and homologous recombination (HR) pathways, including XPA, OGG1, and BRCA1, are downregulated (Fedeles and Essigmann, 2018[11]; Liu et al., 2018[33]). The consequence is persistent DNA adducts and progressive accumulation of secondary mutations, generating a mutator phenotype that drives malignant progression. This represents a feed-forward mechanism: DNA damage would normally activate repair, yet AFB1 simultaneously undermines repair capacity.

### 3.2 Accelerators: oxidative stress, epigenetics, and autophagy

#### 3.2.1 Oxidative stress

Reactive oxygen species (ROS) generation constitutes the core mechanism through which AFB1 amplifies its carcinogenic effects, serving both as a direct source of macromolecular damage and as a signaling hub that activates pro-inflammatory and pro-survival pathways (Tian et al., 2023[61]; Wu et al., 2022[72]; Zhang et al., 2023[84]). ROS originate from redox cycling of AFB1 metabolites, mitochondrial dysfunction, and nicotinamide adenine dinucleotide phosphate hydrogen (NADPH) oxidase activation. Uncontrolled oxidative bursts deplete glutathione (GSH) and reduce superoxide dismutase (SOD) and catalase activities (Wu et al., 2022[72]; Zhang et al., 2023[84]).

Acting as second messengers, ROS activate the NF-κB pathway through IκBα degradation and nuclear translocation of p65, which transactivates genes encoding pro-inflammatory cytokines (IL-1β, TNF-α), anti-apoptotic factors, and proliferation initiators (Hou et al., 2018[16]; Zhang et al., 2024[83]). Sustained NF-κB activation converts acute oxidative injury into chronic inflammation, a well-established driver of hepatocarcinogenesis.

Importantly, AFB1-induced oxidative damage arises not only from direct ROS attack but also from secondary inflammation triggered by cellular debris following cell death. Fishbein and colleagues (Fishbein et al., 2020[12]) demonstrated that AFB1-generated apoptotic fragments induce an eicosanoid and cytokine storm in macrophages through upregulation of COX-2, soluble epoxide hydrolase (sEH), and endoplasmic reticulum stress genes (BiP, CHOP, PDI), promoting HCC growth. Concurrent blockade of COX-2 and sEH abrogated this storm, enhanced fragment phagocytosis, and suppressed tumor progression. This reveals a unique pathway through which AFB1 triggers sterile inflammation via apoptotic debris.

AFB1-induced oxidative stress also profoundly impacts lipid metabolism. Yang and colleagues (Yang et al., 2023[79]), employing metabolomics and lipidomics, demonstrated that AFB1 and its metabolite AFM1, alone and in combination, induce lipid metabolic disturbances in NCM460 cells, with combined exposure producing broader perturbations. Glycerophospholipid metabolism, fatty acid degradation, and propionate metabolism were the primary affected pathways. AFB1 predominantly affected cardiolipin (CL) and triglycerides (TAG), whereas AFM1 primarily impacted CL and phosphatidylglycerol. Lipid peroxidation products further amplify ROS signals, establishing a positive feedback loop.

Multi-omics integration has revealed novel mediators. Chen and colleagues (Chen et al., 2024[7]) performed combined transcriptomic, proteomic, and metabolomic analysis of SW480 cells, coupled with molecular dynamics simulations, revealing that AFB1 significantly enriches pathways related to oxidative stress, fatty acid/lipid metabolism, and glutathione metabolism, and identified sterol carrier protein 2 (SCP2) as a potential key mediator of AFB1 cytotoxicity. SCP2 expression changes were accompanied by elevated ROS, calcium overload, mitochondrial damage, and lipid metabolic disruption.

At the level of antioxidant defense, nuclear factor erythroid 2-related factor 2 (Nrf2) serves as the primary transcriptional regulator. AFB1 downregulates Nrf2 and its downstream targets heme oxygenase 1 (HO-1), NADPH quinone oxidoreductase 1 (NQO-1), and SOD (Tian et al., 2023[61]; Zhang et al., 2023[84]). Furthermore, AFB1 interferes with nuclear receptor signaling. Persico and colleagues (Persico et al., 2023[48]), through computational biology and experimental approaches, revealed that AFB1 competitively antagonizes the vitamin D receptor (VDR) and retinoid X receptor α (RXRα), interfering with vitamin D-mediated transcriptional activation, disrupting VDR and RXRα subcellular localization, and compromising vitamin D functions in immune regulation, cell differentiation, and antioxidant defense. In Caenorhabditis elegans, AFB1 suppresses the forkhead box O (FOXO) transcription factor dauer formation abnormal-16 (DAF-16), disrupting the antioxidant SOD-3 axis and promoting aging and immunotoxicity (Chang et al., 2025[6]), demonstrating the evolutionary conservation of AFB1-induced antioxidant damage.

AFB1 thus cooperatively assaults cellular antioxidant defenses through multiple mechanisms, directly depleting antioxidant enzymes while simultaneously disrupting key transcriptional regulatory pathways including Nrf2, FOXO, and VDR/RXRα, comprehensively dismantling the cellular capacity to cope with oxidative stress.

#### 3.2.2 Epigenetic reprogramming

AFB1 induces profound and persistent epigenetic alterations that lock cells into pro-oncogenic transcriptional states (Ghantous et al., 2021[15]; Li et al., 2023[28]; Michailidis et al., 2025[43]). DNA methylation patterns are extensively remodeled: promoters of tumor suppressor genes, such as p16/cyclin dependent kinase inhibitor 2A (CDKN2A) and ras association domain family 1 isoform A (RASSF1A) undergo hypermethylation and silencing, while global hypomethylation activates oncogenes and transposable elements (Li et al., 2023[28]). In Gambian children, early-life AFB1 exposure was associated with differential methylation at multiple CpG sites in genes involved in inflammation, signaling, and growth pathways (Ghantous et al., 2021[15]), implying that AFB1-induced epigenetic changes may originate early in life and persist throughout the lifespan.

Histone modifications are similarly perturbed. AFB1 alters the balance between active (H3K4me3) and repressive (H3K27me3) marks, modulating chromatin accessibility. Interactions between AFB1 and Epstein-Barr virus may further fine-tune the epigenetic landscape (Michailidis et al., 2025[43]). Non-coding RNAs, including miR-122, miR-34a, and multiple lncRNAs, are dysregulated by AFB1, modulating proliferation, apoptosis, and immune responses (Sohn and Park, 2025[56]; Xu et al., 2022[77]). Chromatin accessibility analyses have revealed that early AFB1 exposure in PK-15 cells significantly alters the chromatin landscape, with bZIP transcription factors, such as activator protein-1 (AP-1), Fos, JunB, Fra2, playing key regulatory roles and novel transcription factors, including brother of regulator of imprinted sites (BORIS), hepatocyte nuclear factor 1β (HNF1β), and activating transcription factor 1 (Atf1), emerging as potential targets (Li et al., 2024[25]).

The convergence of these epigenetic mechanisms indicates that AFB1-induced reprogramming is not merely a consequence of malignant transformation but a driver of it, providing a long-lasting memory of toxicity that persists beyond the initial insult.

#### 3.2.3 Autophagic dysfunction

Autophagy is a critical process by which cells degrade damaged organelles and proteins to maintain homeostasis. AFB1 interferes with autophagy through inhibition of the mechanistic target of rapamycin kinase/ unc-51 like autophagy activating kinase 1 (mTOR/ULK1) pathway, leading to autophagic flux blockade and accumulation of dysfunctional mitochondria and toxic protein aggregates (Li et al., 2026[30]; Wu et al., 2022[72]). This collapse of housekeeping functions creates conditions favorable for malignant transformation. However, autophagy plays a dual role in AFB1 hepatocarcinogenesis and it can also serve as a protective mechanism limiting damage (Li et al., 2026[30]). This duality positions autophagy as a nuanced regulatory node. Strategies aimed at restoring autophagy (e.g., rapamycin) represent an underexplored frontier.

### 3.3 The soil: immunosuppressive microenvironment remodeling

#### 3.3.1 PD-L1 upregulation and T cell exhaustion

A critical recent advance is the discovery that AFB1 actively promotes immune evasion through upregulation of PD-L1 on hepatocytes (Mary et al., 2024[41]). This effect is mediated, at least in part, by AHR signaling, providing a direct molecular link between AFB1 exposure and immune checkpoint activation. The resultant T cell exhaustion, characterized by impaired proliferation, reduced cytokine production, and diminished cytotoxicity, creates a microenvironment permissive for immune evasion. This finding carries direct therapeutic implications: AFB1-associated HCC may be particularly amenable to immune checkpoint blockade.

#### 3.3.2 Macrophage polarization and regulatory T cell dysregulation

AFB1 skews macrophages toward the pro-tumorigenic M2 phenotype, characterized by production of immunosuppressive cytokines such as IL-10 and TGF-β that promote angiogenesis and tissue remodeling (Hou et al., 2018[16]; Najafi et al., 2020[44]). AFB1, in combination with the co-occurring mycotoxin fumonisin B1 (FB1), alters the Treg/Th17 differentiation balance in an AHR-dependent manner, with the mixture producing greater enhancement of the Treg/Th17 ratio and immunosuppression than either toxin alone (Mary et al., 2024[41]).

The immunosuppressive effects of AFB1 are particularly pronounced in vulnerable populations. Among Kenyan women living with HIV, plasma AFB1 concentration was associated with virologic failure following antiretroviral therapy (Tonui et al., 2025[63]). In Ugandan women, higher AFB1 exposure increased both the detection and persistence of oncogenic HPV 16 and HPV 18 (Tong et al., 2025[62]).

#### 3.3.3 Cross-species immunomodulation

The immunotoxicity of AFB1 spans species boundaries. In neonatal cattle and camels, naturally occurring AFB1 levels induce neutrophil apoptosis, which colostrum fails to prevent (Najafi et al., 2020[44]). In chickens, AFB1 triggers immunotoxicity through mRNA-miRNA pathway crosstalk in immune organs (Xu et al., 2022[77]).

### 3.4 The seed and soil integrative model

AFB1 hepatocarcinogenesis arises from simultaneous perturbations across three levels:

1. Genomic level (the seed): Genotoxic injury initiates carcinogenesis through DNA adduct formation and oncogenic mutations, particularly TP53 codon 249, with impaired DNA repair further exacerbating genomic instability.

2. Cellular homeostatic level (the accelerators): Oxidative stress drives the transition from acute injury to chronic inflammation. Epigenetic reprogramming locks cells within pro-oncogenic transcriptional profiles. Autophagic dysfunction enables the buildup of damaged macromolecules and organelles.

3. Microenvironmental level (the soil): PD-L1 upregulation, T cell exhaustion, M2 macrophage polarization, and Treg dysregulation remodel the immunosuppressive microenvironment.

These dimensions do not function in isolation. Genotoxic injury triggers inflammatory pathway activation. Inflammatory mediators impair DNA repair capacity and elicit epigenetic alterations. Epigenetic modifications upregulate pro-inflammatory and immune checkpoint gene expression. Immunosuppression facilitates the survival and progression of mutated cells. AHR sits at the core of this network and serves as a bridge linking metabolic activation to immune modulation (Figure 6(Fig. 6)).

## 4. Expanding the Scope of Synergistic Carcinogenesis: From Intrahepatic Metabolism to Systemic Perturbation

The synergistic carcinogenicity of AFB1 with HBV/HCV has been repeatedly confirmed by epidemiological studies (Jin et al., 2023[20]; Xing et al., 2025[76]). Yet the meaning of synergy is expanding with ongoing research, extending from intrahepatic metabolic reprogramming to encompass the gut-liver axis, systemic immunosuppression, and combined exposure to multiple mycotoxins.

### 4.1 Epidemiological evidence

The epidemiological evidence for synergistic HCC risk driven by combined AFB1 exposure and chronic HBV infection stands as one of the most compelling examples of gene-environment interaction. Individuals exposed to both agents face a 30- to 60-fold higher HCC risk than those exposed to either alone (Jin et al., 2023[20]; Xing et al., 2025[76]).

### 4.2 The multifaceted actions of HBx

The X protein of HBV (HBx) is a pleiotropic viral oncoprotein that potentiates AFB1 genotoxicity through multiple mechanisms. HBx upregulates CYP1A2 and CYP3A4, enhancing AFB1 activation to AFBO (Jin et al., 2023[20]). HBV-infected hepatocytes thus become hotbeds for AFB1 activation. In tree shrew models, combined AFB1 and HBV exposure led to inactivation of the FTCD-AS1-PXR-MASP1 axis, suppressing PXR signaling and exacerbating liver injury and carcinogenic risk (Xing et al., 2025[76]). HBx impairs DNA repair capacity, permitting persistence of AFB1-DNA adducts and increasing the probability of mutation fixation. The chronic inflammation driven by persistent immune responses and pro-inflammatory cytokines in HBV infection forms a positive feedback loop with AFB1-induced oxidative stress, creating a perfect storm for HCC development (Jin et al., 2023[20]; Xing et al., 2025[76]).

### 4.3 Indirect contributions of multi-organ toxicity

The extrahepatic toxicity of AFB1 may indirectly exacerbate HBV-associated HCC through systemic pathways. AFB1, activated by intestinal CYP1A1, causes barrier disruption (Gerdemann et al., 2023[14]), allowing lipopolysaccharide (LPS) translocation into the bloodstream, which activates hepatic Kupffer cells through TLR4/NF-κB, amplifying inflammation in the HBV context (Zhang et al., 2024[83]). This constitutes an indirect pathway of AFB1 intestinal injury-LPS translocation to liver-inflammatory amplification-HBV synergistic carcinogenesis. AFB1 induces immune cell apoptosis and dysfunction in the spleen, bursa of Fabricius, and bone marrow (Najafi et al., 2020[44]; Xu et al., 2022[77]). In the context of HBV infection, this may compromise the ability of CD8⁺ T cells to clear virus and tumor cells. Data from Kenyan women with HIV further support this perspective (Tonui et al., 2025[63]). HBx can reach extrahepatic tissues through exosomes or the circulation (Jin et al., 2023[20]). AFB1 itself impairs testicular germ cell development through Ras/PI3K/Akt (Zhang et al., 2023[81][84]) and induces renal tubular epithelial cell apoptosis via the RIPK4-p53 axis (Ma et al., 2025[35]). AFB1-HBV synergy should not be reduced to a simplistic two factors adding up within the liver. It represents a process whereby AFB1 systemically disrupts immune and metabolic homeostasis through multi-organ toxicity, creating a systemic environment permissive for HBV-driven hepatocarcinogenesis.

### 4.4 Modulatory effects of co-occurring mycotoxins

Populations in HBV-endemic regions are typically exposed to multiple foodborne contaminants simultaneously. The modulatory effects of co-occurring toxins on AFB1-HBV synergy have received remarkably little attention. Willoquet and colleagues (Willoquet et al., 2024[71]) found that deoxynivalenol (DON), through inhibition of ribosomal function, reduces CYP1A2 and CYP3A4 protein levels/activity, diminishing AFB1 biotransformation to AFBO and consequently reducing AFB1 genotoxicity. However, DON itself possesses immunotoxic properties that may enhance HBV risk from another direction. The net effect represents the algebraic sum of multiple actions and remains unclear. Ochratoxin A (OTA) and AFB1 both activate AHR (Hou et al., 2018[16]; Shin et al., 2019[53]). In the HBV context, sustained AHR activation not only enhances AFB1 activation but also upregulates PD-L1 (Mary et al., 2024[41]), promoting T cell exhaustion. Combined OTA and AFB1 exposure may, through additive effects on the AHR pathway, produce immunosuppression exceeding that of AFB1 alone. Fumonisin B1 (FB1) combined with AFB1 enhances hepatocyte toxicity, exacerbating oxidative stress and DNA damage through induction of CYP activity and arachidonic acid metabolism (Mary et al., 2017[40]). FB1, through inhibition of ceramide synthesis, alters sphingolipid metabolism, potentially interfering with pre-existing lipid reprogramming in HBV-infected hepatocytes (Mary et al., 2024[41]). The majority of epidemiological studies consider only AFB1 exposure in isolation. Against the backdrop of climate change-driven shifts in toxin contamination profiles, future HCC risk assessment must incorporate the concept of mycotoxin mixtures. The dual-hit paradigm, whereby AFB1 provides genotoxic injury while HBV enhances metabolism, suppresses repair, and provides an inflammatory microenvironment, remains the foundational model for understanding synergy (Kumari et al., 2026[21]). However, emerging evidence suggests that: (1) intervention targets should expand to include the intestinal barrier, with probiotics and mucosal protectants potentially reducing HCC risk indirectly by mitigating AFB1-induced intestinal injury; (2) immunomonitoring of HBV carriers should incorporate AFB1 exposure assessment; and (3) risk assessment should account for combined exposure to multiple mycotoxins (Figure 7(Fig. 7)).

## 5. Biomarkers and Risk Assessment

### 5.1 Exposure biomarkers

AFB1-albumin adducts currently constitute the gold standard for assessing chronic AFB1 exposure (Tong et al., 2025[62]; Tonui et al., 2025[63]), reflecting cumulative exposure over weeks to months. Studies employing this biomarker have linked AFB1 exposure to adverse outcomes including impaired HIV suppression and persistent HPV infection. AFB1-treated cells exhibit marked alterations in purine/pyrimidine metabolism, carnitine synthesis, and lipid metabolism (Cao et al., 2025[4]; Wang et al., 2021[67]). The combination of D-optimal mixture design with Orbitrap Fusion mass spectrometry has enabled multi-pathway quantification of AFB1 biosynthetic precursors (Xie et al., 2018[75]).

### 5.2 Effect and susceptibility biomarkers

Detection of circulating tumor DNA bearing the TP53 codon 249 mutation in blood provides a direct measure of early malignant transformation (Fedeles and Essigmann, 2018[11]; Link and Iwakuma, 2017[32]), though sensitivity remains a bottleneck for early detection. AFB1-associated DNA methylation changes in Gambian children suggest that specific methylation patterns may serve as early indicators of exposure effects (Ghantous et al., 2021[15]). Circulating miR-122 and miR-34a represent additional candidates. Polymorphisms in GST (GSTM1/GSTT1 deletion) and CYP450 genes are associated with HCC risk in AFB1-exposed populations (Gerdemann et al., 2023[14]; Thiendedsakul et al., 2022[60]). Serum cytokine profiles, soluble PD-L1 levels, and T cell functional parameters may reflect the degree of immunosuppression and guide immunotherapy selection and monitoring.

### 5.3 Novel markers

Recent years have identified multiple novel host factors. Glycine N-methyltransferase (GNMT) not only participates in AFB1 detoxification but also exerts tumor-suppressive functions through regulation of one-carbon metabolism and the p53 pathway, with its downregulation correlating with hepatocarcinogenesis (Chen et al., 2019[8]). Epigenetic silencing of CDKN2A (encoding p16INK4a and p14ARF) represents a common event in AFB1-induced hepatocarcinogenesis, and promoter hypermethylation may serve as a biomarker for early detection and risk stratification (Yang et al., 2026[78]). Major urinary protein 1 (MUP1) was significantly reduced in AFB1-exposed type 1 diabetic mice (Tsai et al., 2017[64]).

### 5.4 Next-generation risk prediction models

The complexity of AFB1 hepatocarcinogenesis demands multi-factorial integrated risk assessment. An ideal model should incorporate: (1) exposure (AFB1-albumin adducts, urinary AFM1, dietary data); (2) host genetics (CYP/GST polymorphisms); (3) epigenetics (DNA methylation, miRNAs); (4) infection status (HBV/HCV serology, viral load); (5) immune parameters (cytokines, PD-L1, T cell subsets); and (6) clinical indicators (liver function, fibrosis, imaging). Machine learning approaches can capture non-linear interactions across these dimensions to deliver individualized risk prediction (Figure 8(Fig. 8)).

## 6. Intervention and Control Strategies

Prevention and control of AFB1-associated HCC span the entire continuum from farm to clinic. The following sections address these strategies hierarchically: source control, chemoprevention, biocontrol, and clinical therapy.

### 6.1 Source control and biosynthetic regulation

The most effective strategy remains primary prevention, preventing AFB1 entry into the food chain. Specific measures include deployment of resistant crop varieties, application of biocontrol agents such as non-toxigenic A. flavus strains (e.g., Afla-Guard), optimization of storage conditions, and climate-adaptive agricultural practices (Rushing and Selim, 2019[50]). Emerging technologies including cold plasma and nitric oxide fumigation (Chen et al., 2025[9]; Mahmoud et al., 2025[37]) offer novel tools for detoxification. However, the efficacy of these measures is substantially constrained by existing agricultural practices and the controllability of climatic conditions, necessitating coordination with other intervention modalities.

On a parallel technological front, targeting key regulatory factors in the AFB1 biosynthetic pathway to disrupt toxin production at the fungal metabolic level has yielded important advances. Among transcription factors, deletion of the C_2_H_2_-type transcription factor AflZKS3 completely abolishes AFB1 production while concurrently reducing strain pathogenicity (Liang et al., 2022[31]). Histone deacetylase HosA directly binds AFB1 cluster genes to positively regulate their expression (Lan et al., 2019[23]; Yang et al., 2023[79]), while AfldrnA participates in regulating the conidia-sclerotia developmental balance (Abdo-Elgabbar et al., 2026[1]). At the signaling pathway level, the interaction between Fus3-MAPK and the Snf1/AMPK subunit Gal83 is involved in regulating AFB1 biosynthetic substrates (Ma et al., 2024[34]). Benzoylation of the CBS protein (catalyzed by GcnE) influences fungal development and virulence (Wu et al., 2026[74]).

Biocontrol microorganisms have also demonstrated considerable potential. The yeast Wickerhamomyces anomalus WRL-076 downregulates AFB1 cluster genes and conidiation regulatory genes, reducing fungal biomass by 60 % and conidiation by over 95 % (Hua et al., 2019[18]). Of concern, resistance mutations (SdhB H249Y, SdhC G91R) to the widely used SDH1 class of fungicides have been found to be associated with AFB1 production (Masiello et al., 2020[42]), highlighting the need to weigh potential consequences in disease management strategy selection. Additionally, a site-specific integration system based on the sdh2 locus (Tao et al., 2020[58]) provides a reliable genetic tool for functional studies of the aforementioned regulatory factors.

### 6.2 Chemoprevention and dietary intervention

Given the dual central role of AHR in AFB1 metabolic activation (CYP induction) and immune regulation (PD-L1 upregulation), it has emerged as the golden target for chemoprevention. Ideal AHR antagonists or selective modulators should simultaneously reduce AFB1 activation and prevent aberrant PD-L1 upregulation (Budin et al., 2021[3]; Mary et al., 2024[41]). The natural product indole-3-carbinol (I3C) and its derivative 3,3'-diindolylmethane (DIM) exemplify this approach, acting through modulation of AHR and CYP activity to prevent AFB1-induced hepatocarcinogenesis (Williams, 2021[70]). However, AHR also plays indispensable physiological roles in maintaining immune homeostasis and intestinal barrier integrity, necessitating the use of highly selective modulators rather than complete antagonists to avoid disruption of normal AHR function.

In the realm of antioxidant and autophagy modulation, a range of natural bioactive compounds have demonstrated potential to mitigate AFB1 toxicity, including astaxanthin (Tian et al., 2023[61]), curcumin (Li et al., 2021[29]; Zhang et al., 2023[86]), baicalin (Wen et al., 2024[69]), taurine (Wu et al., 2022[72]), resveratrol (Pauletto et al., 2021[47]), and [6]-gingerol (Furlan and Bren, 2020[13]). The mechanisms of these compounds primarily involve activation of the Nrf2 pathway, efficient ROS scavenging, JNK inhibition, and mitochondrial protection. Compared with these broad-spectrum antioxidants, selective ROS scavengers (e.g., Mito-TEMPO) and autophagy activators (e.g., rapamycin) represent more targeted and precise intervention strategies (Li et al., 2026[30]). Additionally, β-carboline alkaloids have been reported to exhibit activity against chemically induced HCC (Saha et al., 2026[51]).

Dietary components also warrant attention for their intervention potential. Salvia miltiorrhiza polysaccharides attenuate AFB1-induced jejunal injury through remodeling of gut microbiota, enhancement of antioxidant capacity, and suppression of the TLR4/MyD88/NF-κB signaling pathway (Zhang et al., 2024[83]). Polyphenols and betalains from red beetroot influence AFB1 bioavailability in Caco-2 cell models, suggesting their potential to reduce toxin absorption (Paula et al., 2025[46]). Fermented Houttuynia cordata extract exhibits inhibitory effects against both hepatic and colonic precancerous lesions through anti-inflammatory, anti-genotoxic, and phase I/II enzyme-modulating activities (Singai et al., 2024[54]). Isochlorogenic acid A has been shown to antagonize combined mycotoxin-induced mitochondrial damage (Lai et al., 2024[22]), offering a novel candidate molecule for chemoprevention in the context of combined exposure.

### 6.3 Adsorbents and microbial/enzymatic interventions

Distinct from the aforementioned host metabolic pathway-targeting strategies, adsorbent-based and microbial/enzymatic interventions provide detoxification routes that do not depend on host metabolism, acting to bind or degrade AFB1 before absorption.

In the realm of plant-based adsorbents, kale and lettuce residues have been found to remove 83.7-93.6 % of AFB1 in simulated gastrointestinal environments, with mechanisms involving hydrophobic interactions, hydrogen bonding, electrostatic attraction, and formation of AFB1-chlorophyll complexes (Vazquez-Duran et al., 2021[65]), offering new possibilities for developing biosorbents from food processing byproducts.

Microbial interventions similarly show broad promise. The probiotic Bacillus amyloliquefaciens suppresses AFB1-induced hepatocyte pyroptosis through activation of the Nrf2/HO-1 antioxidant pathway (Wang et al., 2025[66]). Bioactive substances produced by Achromobacter xylosoxidans ZJS2-1 induce Aspergillus apoptosis through targeting of SrbA, achieving 99 % inhibition of AFB1 production (Sun et al., 2024[57]). Of particular note, the contribution of lactic acid bacteria to AFB1 removal through physical binding mechanisms (16-71 %) far exceeds that of enzymatic degradation (0-18 %) (Lemmetty et al., 2025[24]), suggesting that binding capacity, rather than degradation capacity, should serve as the key criterion for screening effective probiotic strains.

In the field of enzymatic degradation, extracellular enzymes from B. amyloliquefaciens ZG08 degrade 80.93 % of AFB1 within 72 hours (Shi et al., 2024[52]). Dipeptidyl peptidase III from A. terreus (ADPP III) converts AFB1 to aflatoxin D1 (AFD1) with substantially reduced toxicity (Zhang et al., 2024[82]). Luteimonas sp. CW574 possesses the dual capacity to degrade both AFB1 and ochratoxin A (Zhang et al., 2025[85]), offering unique advantages in combined toxin contamination scenarios. DypB peroxidase transforms AFB1 into four less toxic products (Mangini et al., 2024[38]), further enriching the toolbox of enzymatic detoxification candidates. Essential oil nanoformulations have also proven effective, with mechanisms involving disruption of fungal membrane integrity, inhibition of ergosterol synthesis, and downregulation of toxin-related genes (Prakash and Raghuvanshi, 2026[49]; Singh et al., 2026[55]), providing possibilities for developing integrated antifungal-detoxification formulations.

### 6.4 Therapeutic strategies for precision and immunotherapy

AFB1-associated HCC displays molecular features including high mutational burden, DNA repair defects, and overactivation of the AHR/PD-L1 pathway-characteristics that present unique opportunities for targeted therapy. The demonstration that AFB1 induces PD-L1 upregulation provides a strong mechanistic rationale for immune checkpoint blockade (Mary et al., 2024[41]; Tong et al., 2025[62]; Tonui et al., 2025[63]), suggesting that patients with AFB1-associated HCC may be particularly responsive to PD-1/PD-L1 inhibitors. AHR expression level may serve as a predictive biomarker for therapeutic response, with high AHR expressers likely deriving greater benefit from blockade. Additionally, the COX-2/sEH pathway inflammatory storm triggered by AFB1 through apoptotic debris (Fishbein et al., 2020[12]) suggests that combined anti-inflammatory strategies may further enhance therapeutic efficacy.

Several combination approaches warrant prioritization: (1) AHR inhibitors combined with anti-PD-L1, a strategy that simultaneously inhibits AFB1 metabolic activation and PD-L1 upregulation, representing the most promising combination; (2) PARP inhibitors combined with anti-angiogenic agents, as AFB1-induced HR repair deficiency confers intrinsic sensitivity to PARP inhibition (Fedeles and Essigmann, 2018[11]; Liu et al., 2018[33]); (3) antioxidants combined with immunotherapy, as ROS scavenging alleviates inflammation-mediated immunosuppression and improves treatment response; and (4) COX-2/sEH dual inhibitors combined with immunotherapy, with agents such as PTUPB promoting inflammation resolution (Fishbein et al., 2020[12]).

From a total management perspective, a multi-layered prevention and control system covering all stages should be established. Source-level measures include the promotion of resistant varieties and the adoption of biocontrol approaches. Before ingestion, adsorbents and enzymatic degradation technologies can be applied to lower toxin burden. Following ingestion, AHR inhibitors and antioxidants should be administered in a timely manner to achieve chemopreventive effects. At the clinical stage, immunotherapy and combinatorial intervention strategies are to be selected according to molecular features. The construction of such a system depends on continued deep understanding and precise translation of the AFB1 carcinogenic network (Figure 9(Fig. 9)).

## 7. Conclusions and Perspectives

### 7.1 Core conclusions

#### Five core conclusions of this review

1. AFB1-driven hepatocarcinogenesis results from crosstalk between genotoxic injury (seed) and microenvironmental remodelling (soil).
2. AHR serves as the core molecular switch driving AFB1 bioactivation and PD-L1-dependent immune evasion.
3. AFB1-HBV synergistic hepatocarcinogenesis represents a systemic multi-organ process modulated by co-occurring mycotoxins.
4. Multi-layered “farm to clinic” interventions replace isolated single-target countermeasures against AFB1-associated HCC.
5. Integrated biomarkers combined with machine learning enable personalised HCC risk stratification for high-risk populations.

Synthesizing the full body of evidence, five core conclusions emerge. First, AFB1 carcinogenesis is fundamentally the result of synergistic interplay between genotoxic injury and microenvironmental remodeling. The canonical CYP activation-AFBO- DNA adduct-TP53 codon 249 mutation pathway initiates genomic instability, yet this constitutes only the opening chapter. Three cooperating accelerators constitute the persistent drivers of malignant progression. Oxidative stress signaling represented by ROS and NF-κB mediates the shift from acute injury toward chronic inflammation. Epigenetic reprogramming encompassing DNA methylation, histone modifications and non-coding RNAs traps cells in pro-oncogenic transcriptional states. Autophagic dysfunction related to the mTOR/ULK1 pathway allows the progressive accumulation of damaged macromolecules and organelles.

Throughout this process, PD-L1 upregulation, T cell exhaustion, and M2 macrophage polarization collectively remodel the immunosuppressive soil that enables survival and immune evasion of transformed cells. Second, the aryl hydrocarbon receptor (AHR) has been definitively established as the core molecular switch linking metabolic activation and immune modulation. It transcriptionally upregulates CYP enzymes, constituting a feed-forward amplifier of AFB1 toxicity, while simultaneously mediating PD-L1 upregulation to actively promote immune evasion. This dual role positions AHR as the most promising intervention target in AFB1-associated HCC prevention and therapy. Third, AFB1-HBV synergy has expanded from intrahepatic addition to systemic multiplication. Intrahepatically, the HBx protein amplifies AFB1 genotoxicity through three converging mechanisms: metabolic reprogramming, repair suppression, and inflammatory synergy. Beyond hepatic tissues, AFB1 triggers multiple adverse outcomes such as intestinal barrier impairment, systemic immunosuppression, as well as renal and reproductive toxicity. From a systemic viewpoint, these manifestations indirectly heighten hepatocellular carcinoma risk under HBV‑infected conditions. Critically, co-occurring mycotoxins (OTA, FB1, DON) further modulate the intensity of synergy through the AHR axis, lipid metabolism, and CYP activity, rendering real-world exposure risk assessment substantially more complex. Fourth, intervention strategies must transition from single-point to end-to-end approaches. This necessitates the establishment of multi-layered defense lines covering all stages of the food supply chain, from deployment of resistant varieties at the source, through chemoprevention, from pre-ingestion adsorbent and enzymatic detoxification, to precision immunotherapy at the clinical stage. Finally, at the risk assessment level, multi-dimensional integration of exposure biomarkers (AFB1-albumin adducts), effect biomarkers (TP53 mutation, epigenetic marks), and susceptibility biomarkers (GST/CYP genotypes, GNMT, CDKN2A), combined with machine learning algorithms, holds promise for constructing truly individualized next-generation risk prediction models, providing a scientific basis for precise stratification and early intervention in high-risk populations (Figure 10(Fig. 10)).

### 7.2 Future directions

Building on the above insights, several directions merit prioritized pursuit. AHR-targeted intervention urgently requires clinical translation. The key lies in developing highly selective AHR antagonists or selective modulators capable of effectively blocking AFB1‑induced CYP activation and PD‑L1 upregulation while preserving normal AHR functions in intestinal barrier maintenance and immune homeostasis. Such interventions should be systematically evaluated in HBV transgenic models and tree shrews, animal models that more closely approximate human physiology, while clinical feasibility of AHR expression level as a predictive biomarker for immunotherapy response should be actively explored.

The transgenerational epigenetic effects of early‑life exposure represent an urgent unanswered question. The Gambian children study has established an association between AFB1 exposure and differential methylation at multiple CpG sites, raising the concerning possibility that early‑life AFB1 exposure may influence HCC susceptibility in subsequent generations through epigenetic modifications. This hypothesis requires systematic validation through longitudinal birth cohort studies and multi‑generational animal models. 

The role of the gut microbiota in AFB1 metabolism and detoxification warrants deeper investigation. Metagenomic and metatranscriptomic approaches should be applied to systematically dissect the enzymatic pathways of AFB1‑degrading microbial communities, while germ‑free animal and fecal microbiota transplantation experiments can establish causal relationships between microbiota and host protective effects. In HBV‑endemic regions, prospective probiotic intervention trials are needed to assess real‑world efficacy in reducing AFB1‑associated HCC risk.

Spatial omics technologies offer unprecedented resolution for understanding the tumor microenvironment. Spatial transcriptomics, multiplex immunofluorescence, and mass spectrometry imaging can resolve, in tissue situ, the spatial rules governing intercellular interactions within the AFB1‑driven HCC microenvironment, identifying key cellular neighborhoods and ligand-receptor pairs that drive immune evasion and progression, thereby providing spatial‑dimensional evidence for optimizing combination therapeutic strategies.

Accelerating climate change is redrawing the geographic map of AFB1 contamination, necessitating the establishment of prospective risk warning systems. Integration of climate models, crop monitoring data, population exposure biomarkers, and HCC epidemiology into a dynamic of climate-food security-exposure-disease prediction model is needed, with particular attention to spatial overlap between HBV‑endemic regions and expanding AFB1 contamination zones, enabling proactive deployment of adaptive control measures.

Future population‑based cohort studies should abandon single‑toxin thinking in favor of simultaneous detection of multiple mycotoxin exposure levels, constructing composite exposure-virus synergy risk prediction models that reflect the real world, and systematically exploring the contribution of the mycotoxin exposome to HCC etiology. This will provide a more robust evidentiary foundation for public health policy formulation (Figure 11(Fig. 11)).

## Declaration

### Conflict of interest 

The authors declare that they have no competing interests.

### Acknowledgments 

This work was supported by the Joint Research Center of Occupational Medicine and Health, Institute of Grand Health, Hefei Comprehensive National Science Center (Anhui University of Science and Technology) (NO. OMH-2023-14), Medical Special Cultivation Project of Anhui University of Science and Technology (NO. YZ2023H1B005), and the Practice Innovation Training Program for College Students in Anhui Province (No. S202510361030, 202610361041, S202610361086, S202610361087 and S202610361089).

### Artificial Intelligence (AI) - assisted technology 

The authors confirm that no artificial intelligence (AI) technologies, such as large language models (LLMs), chatbots, or AI-based image generators, were utilized in preparing this manuscript. The scientific data were generated without the use of AI tools, and the images, references, results interpretation, and modifications to the scientific conclusions were all conducted manually. All figures in this article were created using BioRender (BioRender.com) through a licensed academic subscription. The authors bear full responsibility for all scientific content, textual writing, data interpretation, and conclusions.

### Availability of data and materials 

Not applicable.

### Consent for publication 

Not applicable.

### Ethics approval and consent to participate 

Not applicable.

### Author's contribution 

Pan Yu: Investigation, resources, funding acquisition, writing-original draft. Jiacheng Li: Investigation, resources, resources, writing-original draft. Weiya Cao: Writing-review & editing, supervision, resources, funding acquisition. Yongchang Cao: Methodology, investigation, data curation, validation. Wenhui Lv and Hanxu Wang: Methodology, investigation, formal analysis. Jiaxiang Zhang: Data curation, validation. Xiaoyu Bai and Shuhui Feng: Formal analysis. All authors have approved the final version of this manuscript.

## Figures and Tables

**Figure 1:**
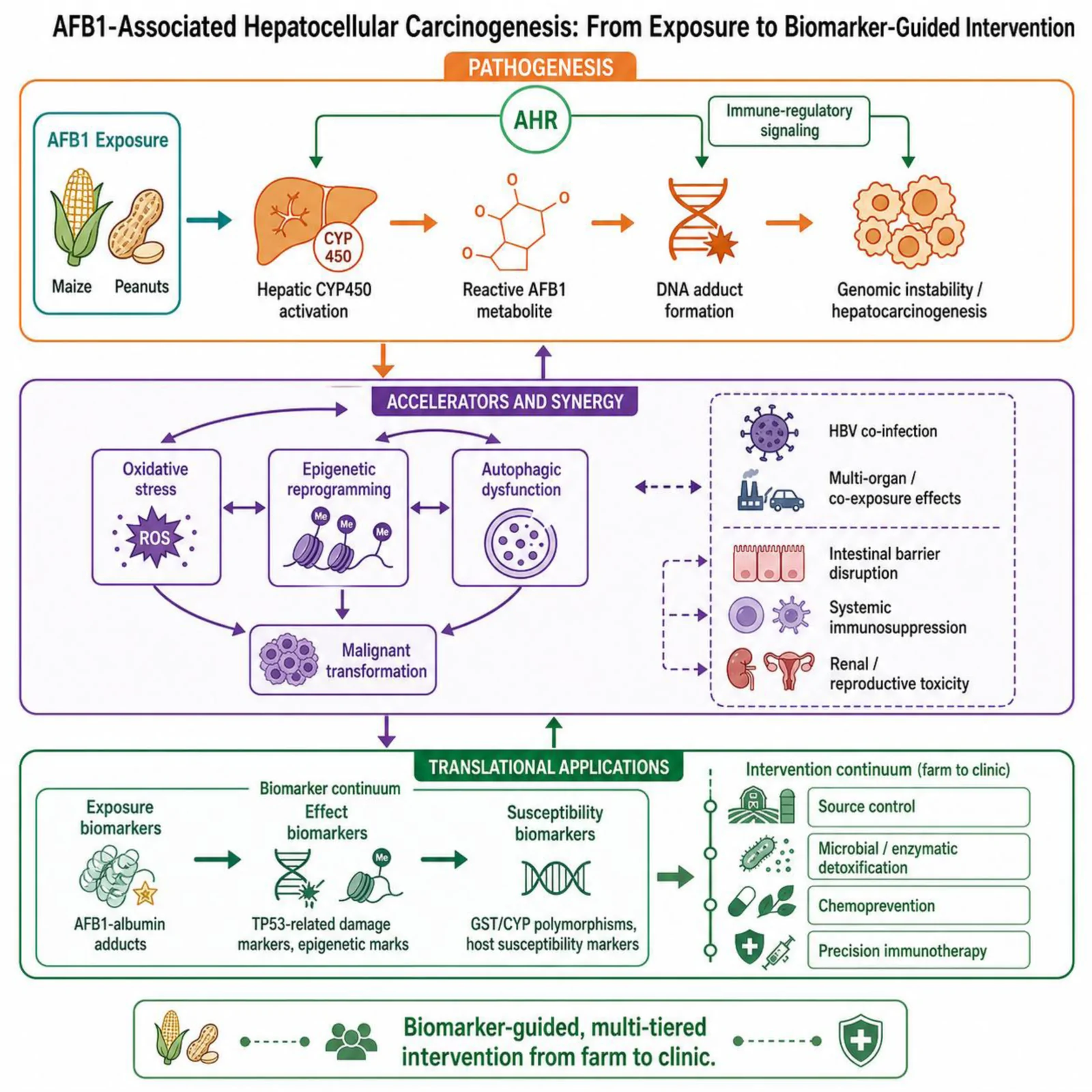
Graphical abstract

**Figure 2:**
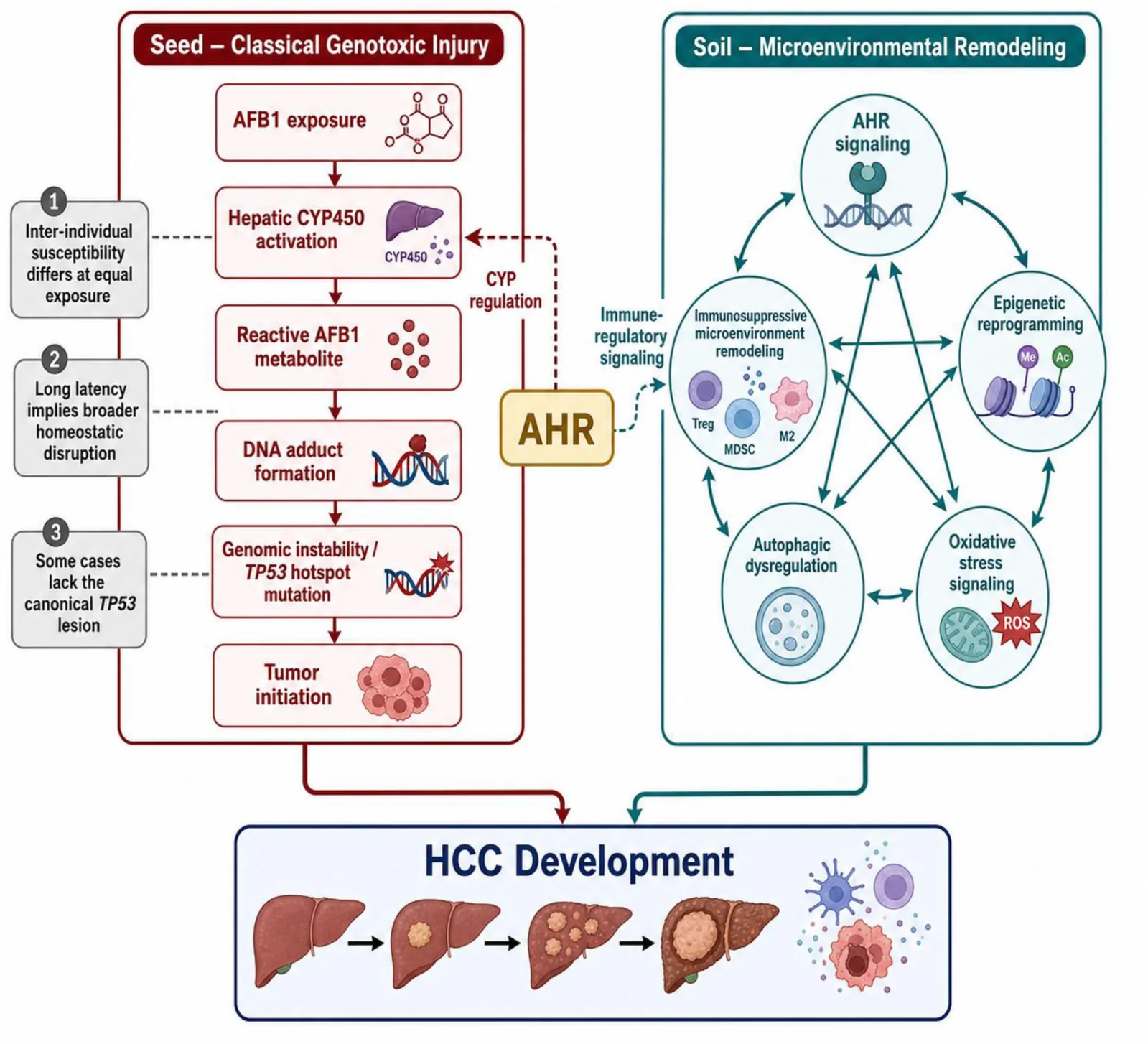
The seed and soil network model of AFB1 hepatocarcinogenesis with AHR as a molecular switch

**Figure 3:**
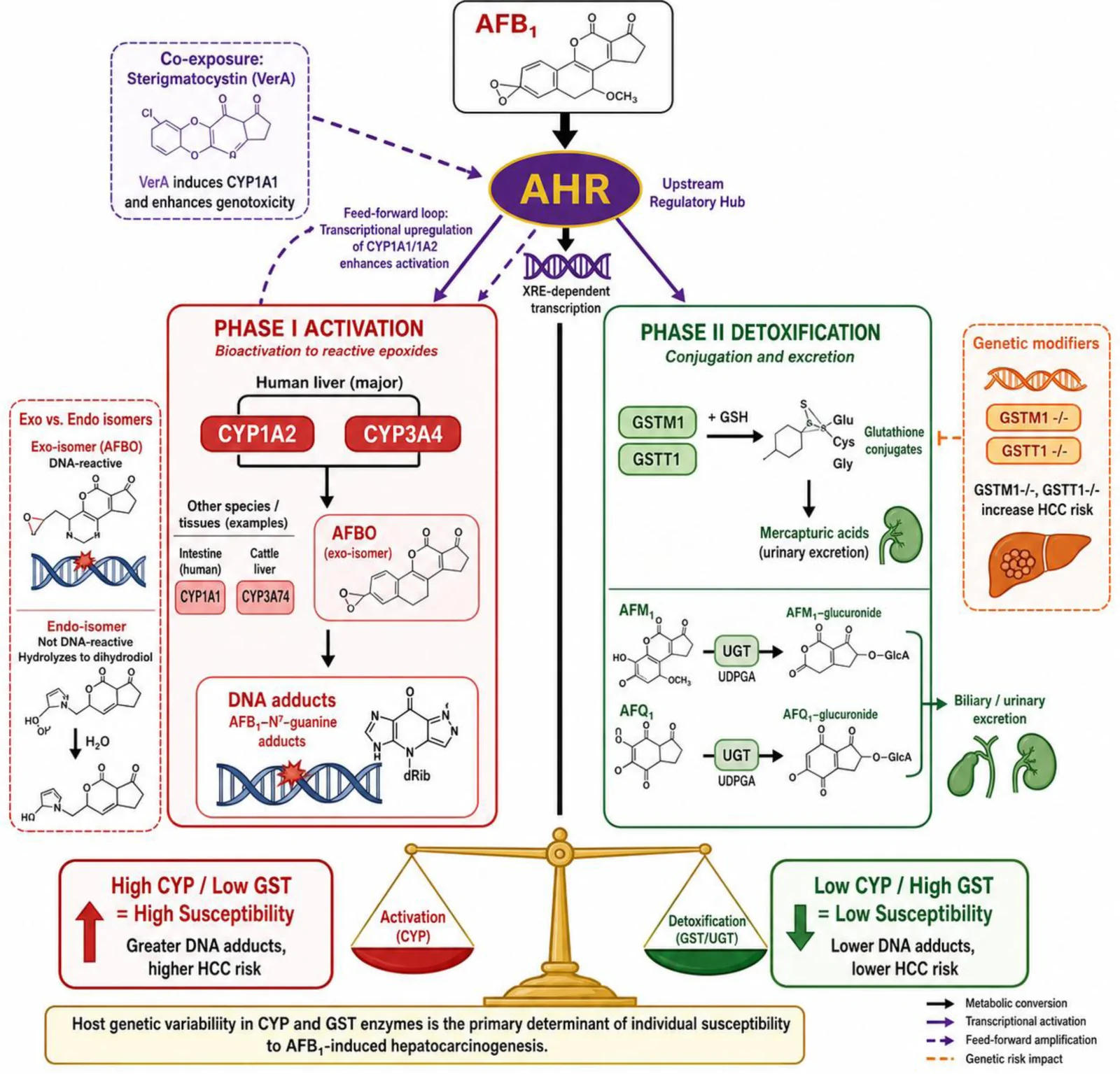
Activation-detoxification balance and the AHR regulatory hub

**Figure 4:**
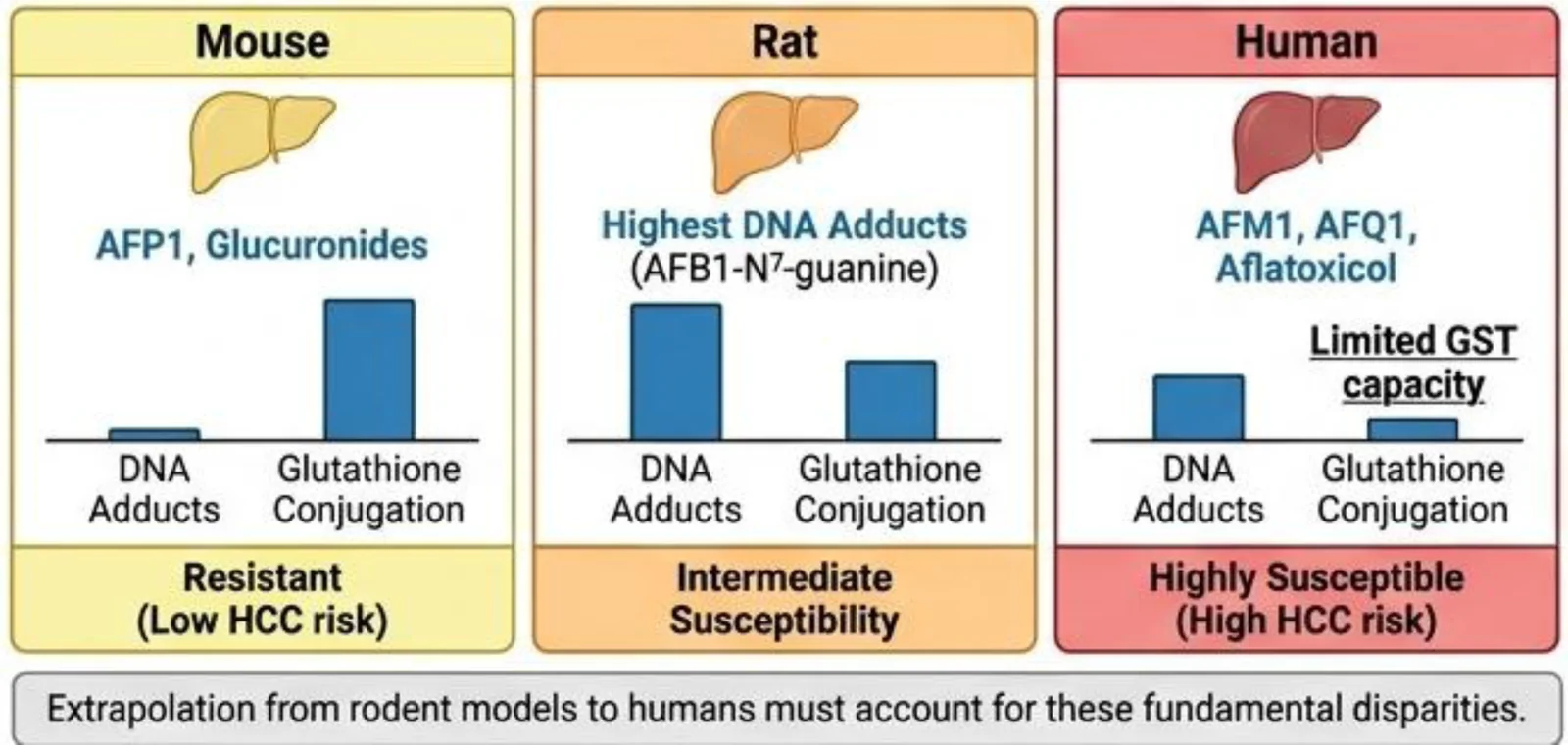
Species-specific metabolic profiles determine differential susceptibility to AFB1 hepatocarcinogenesis

**Figure 5:**
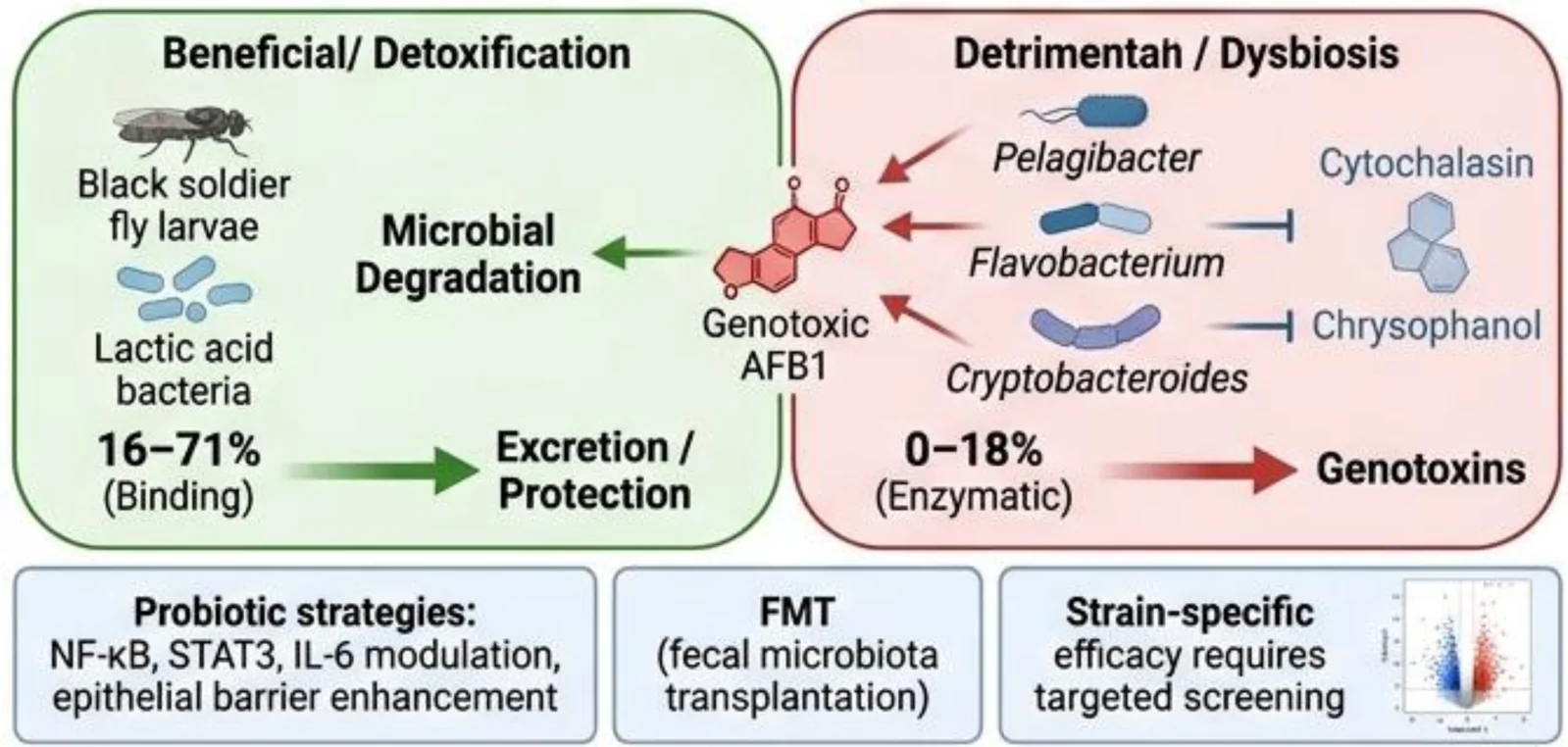
The gut microbiome is involved in AFB1 metabolism through degradation, reactivation, and dysbiosis

**Figure 6:**
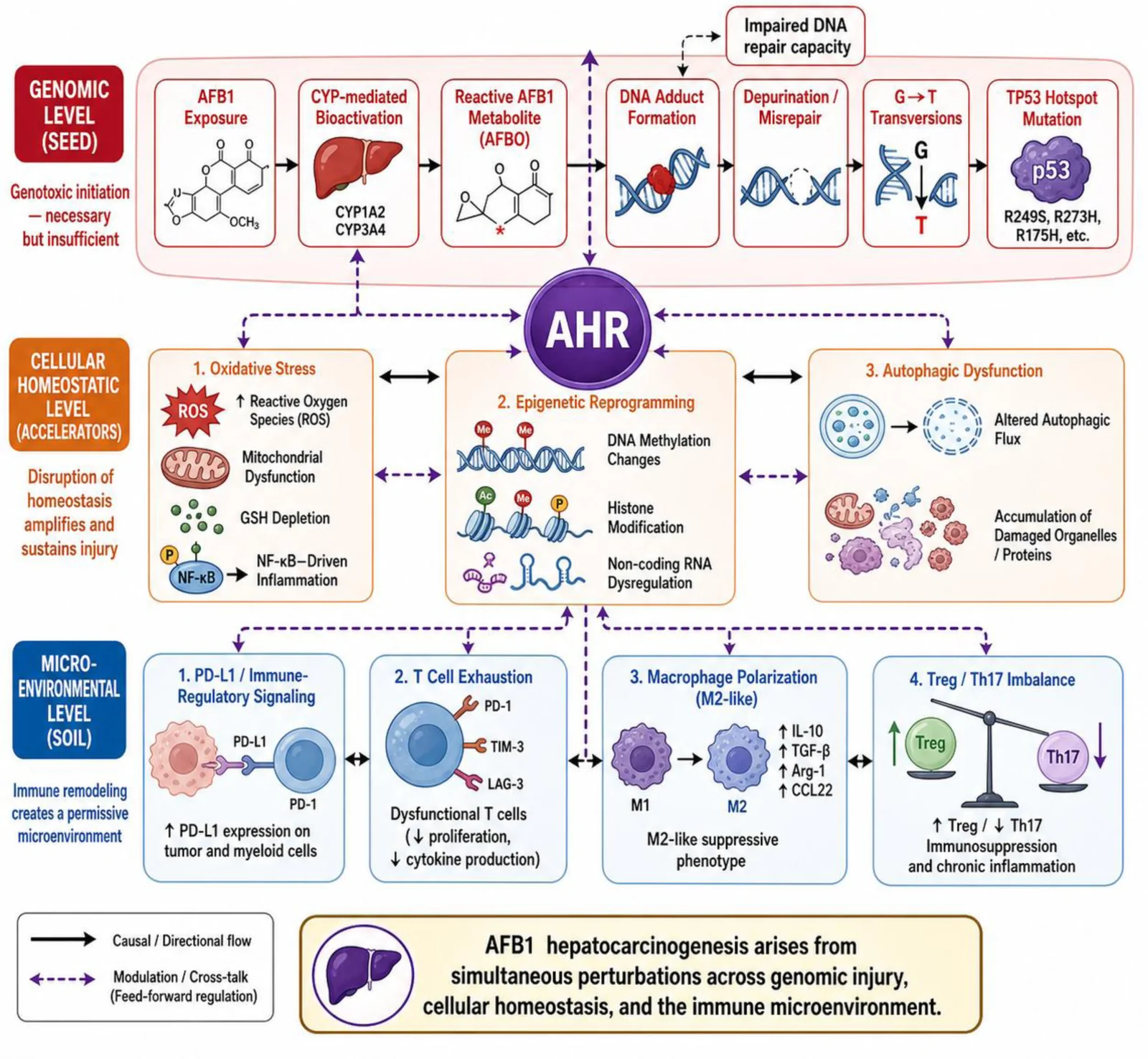
AFB1 hepatocarcinogenesis is governed by an integrated seed and soil three level model with AHR serving as the central bridging node

**Figure 7:**
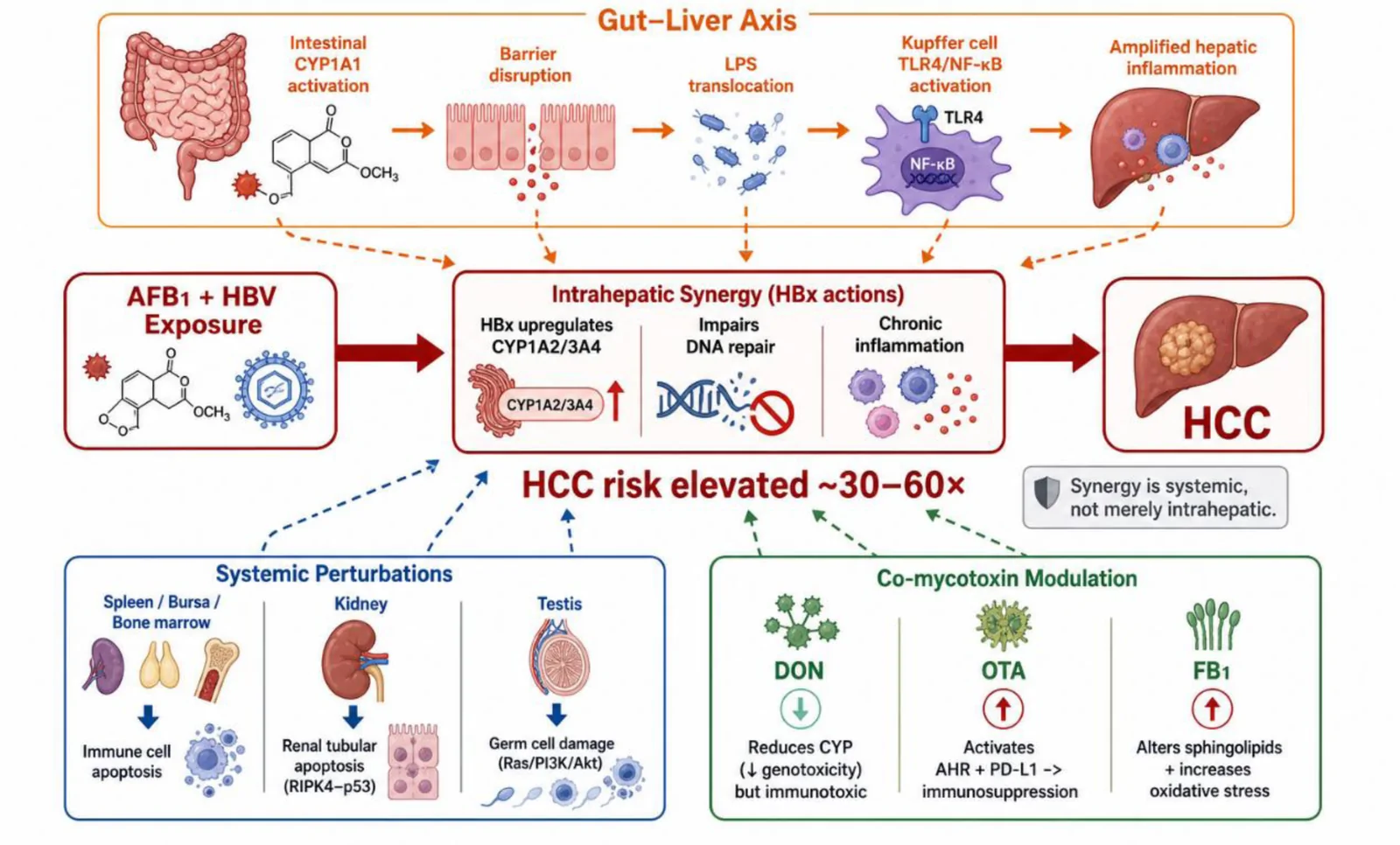
Integrated framework from intrahepatic metabolism to systemic perturbation in AFB1-HBV synergistic carcinogenesis

**Figure 8:**
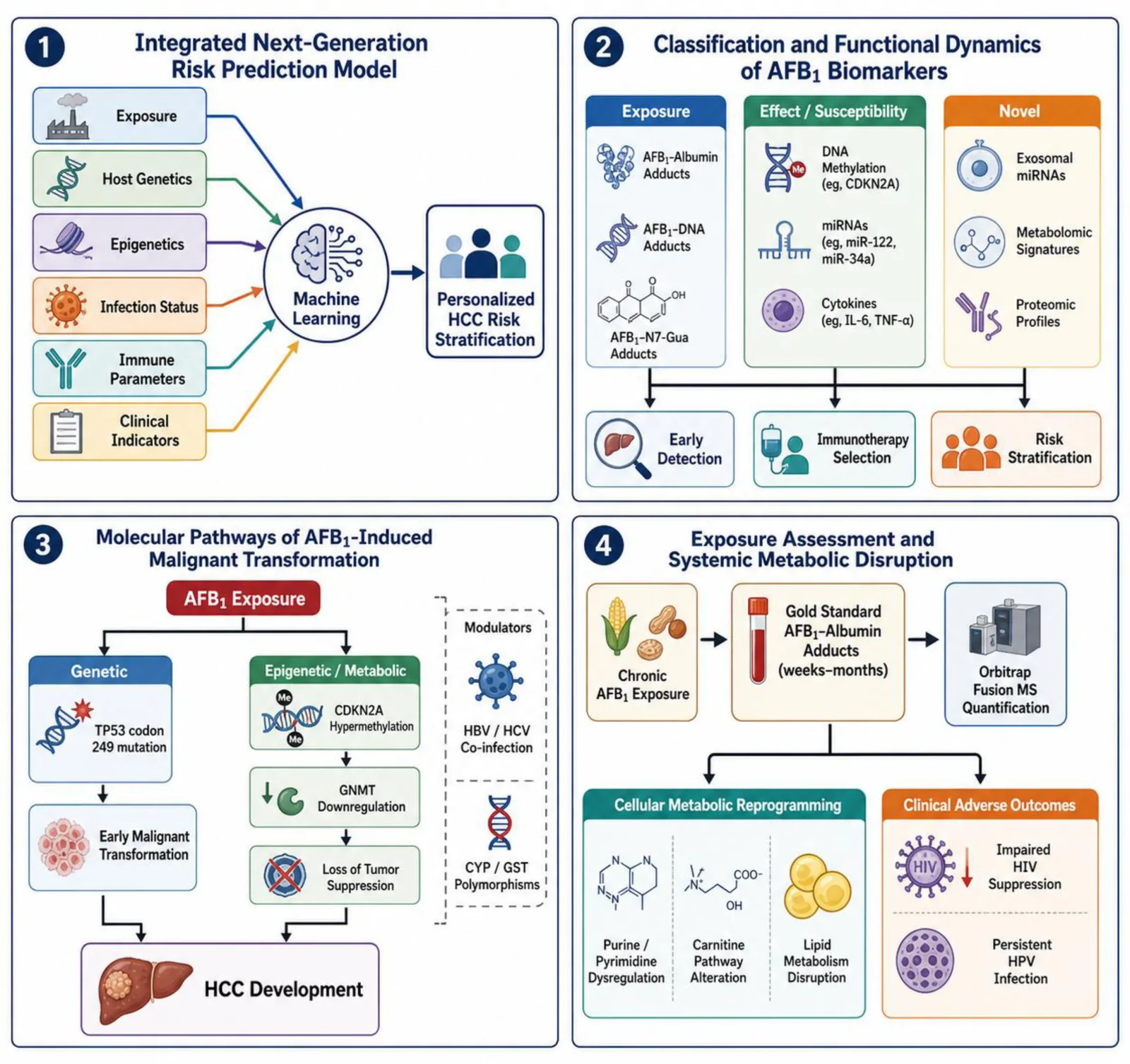
Integrative multidimensional biomarker assessment enables individualized HCC risk prediction in AFB1-exposed populations

**Figure 9:**
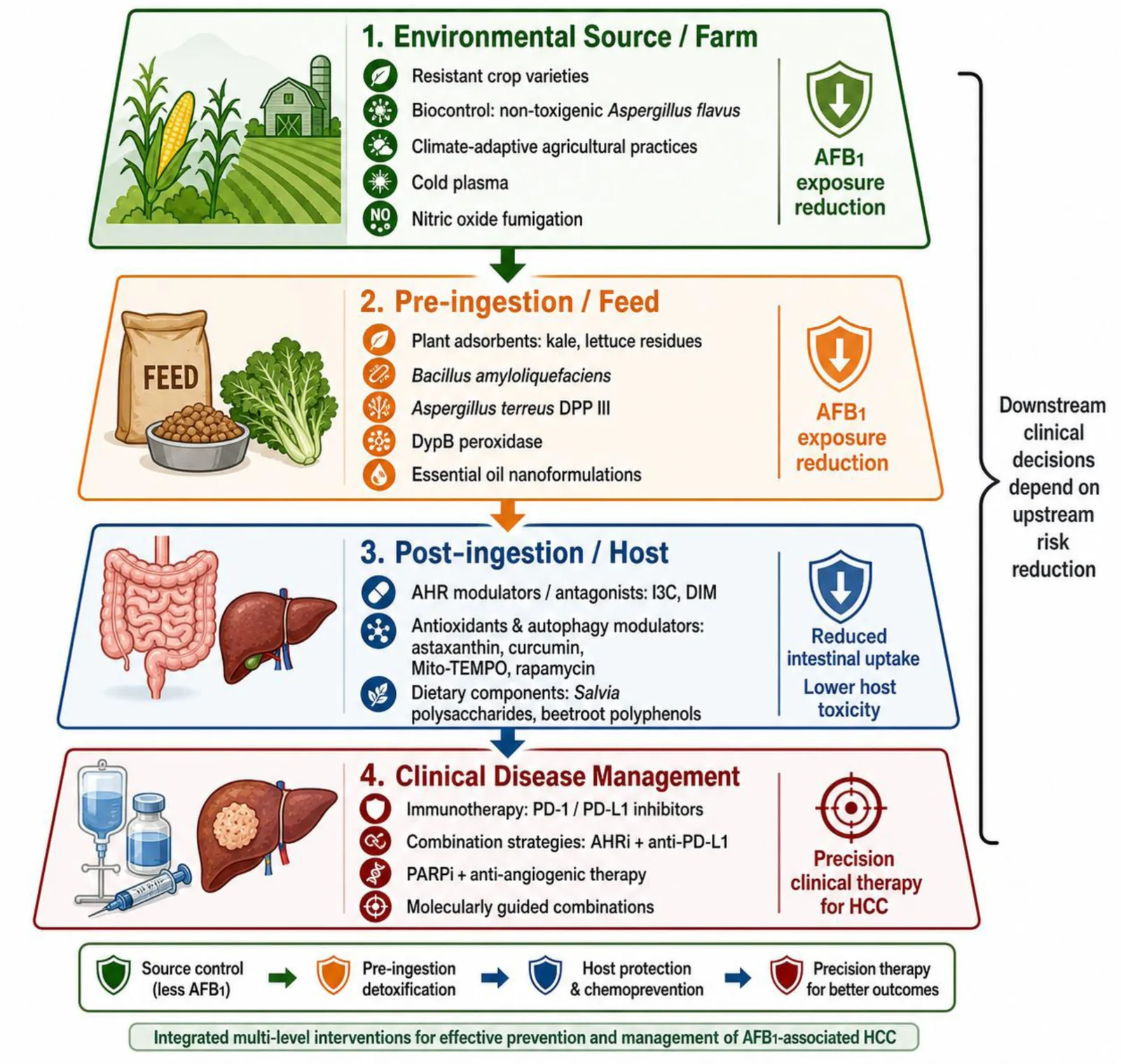
Farm to clinic hierarchical intervention system for AFB1-associated HCC

**Figure 10:**
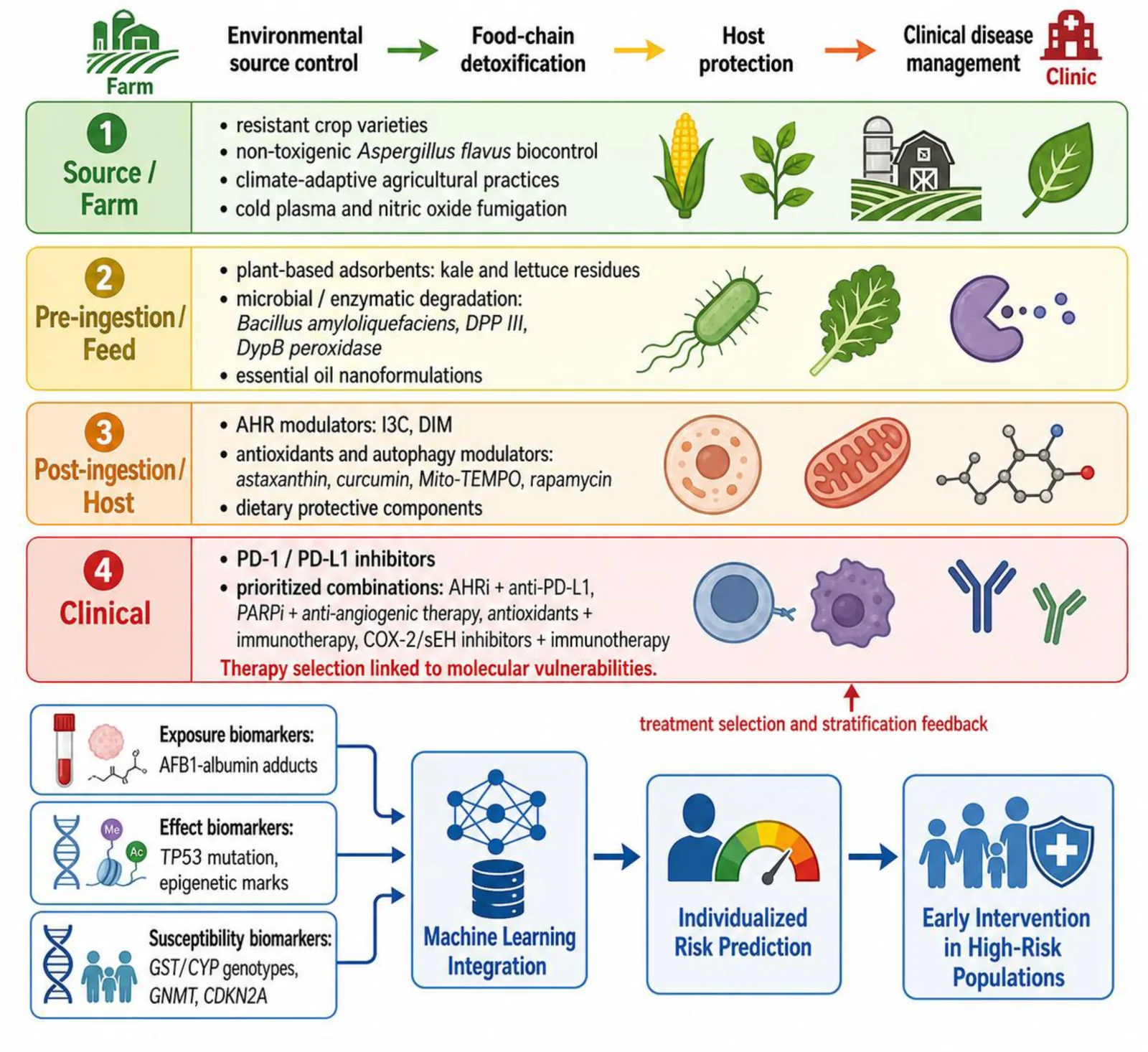
Intervention strategies and next-generation risk assessment for AFB1-associated HCC

**Figure 11:**
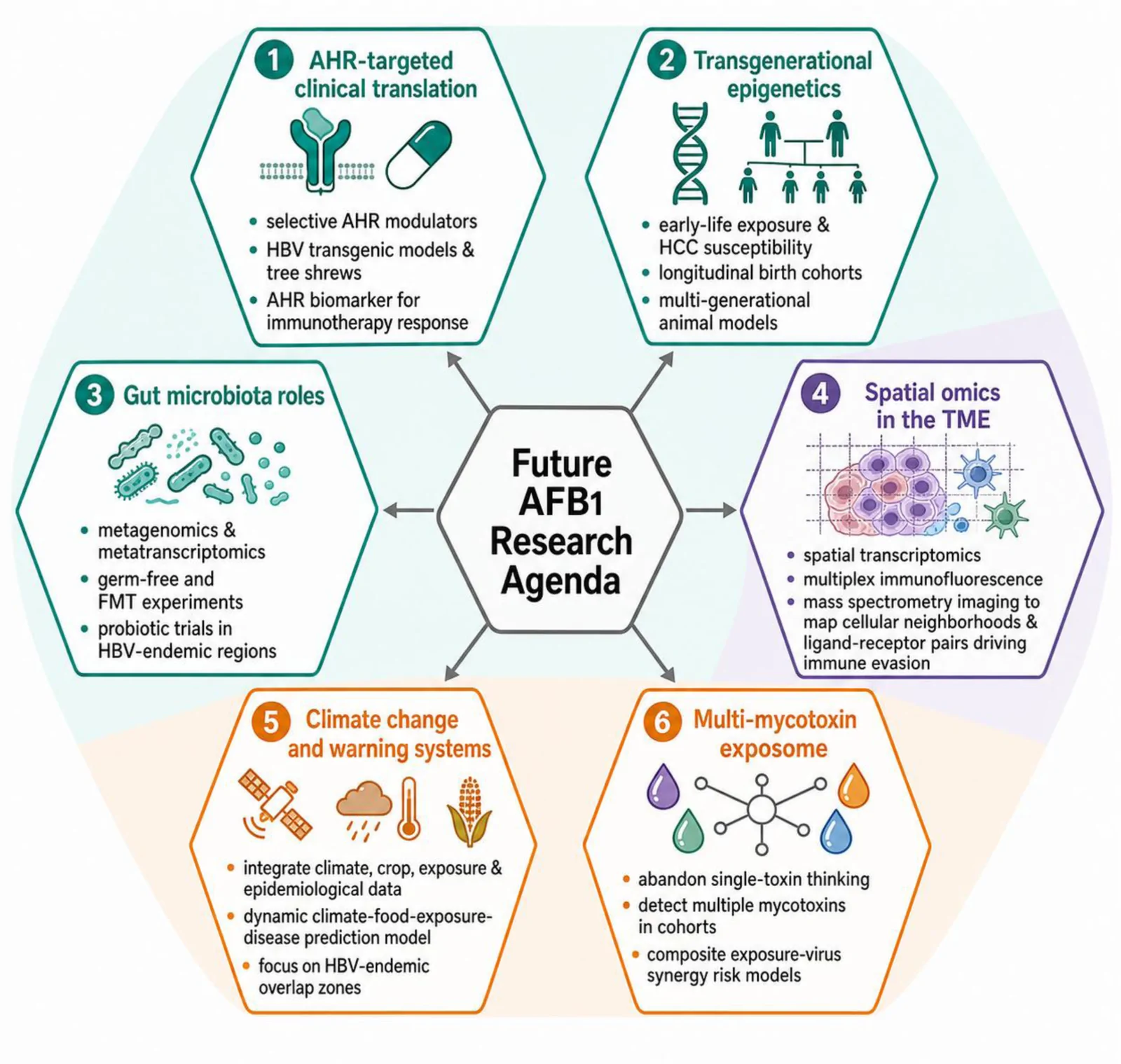
Six prioritized future research directions in AFB1-associated HCC
